# Chromatin state dynamics of autosomes and the B chromosome during spermatogenesis in *Pseudococcus viburni*

**DOI:** 10.1038/s41437-025-00800-x

**Published:** 2025-10-13

**Authors:** Marion Herbette, Laura Ross

**Affiliations:** School of Biological Sciences, Institute of Ecology and Evolution, The University of Edinburgh, Edinburgh, EH9 3FL UK

**Keywords:** Cytogenetics, Imprinting

## Abstract

The mealybug *Pseudococcus viburni* is a plant-feeding insect with a non-Mendelian genetic system known as paternal genome elimination (PGE). In PGE, males eliminate their paternally inherited chromosomes during meiosis, transmitting only the maternal genome to the next generation. This involves genome-wide imprinting, where paternal chromosomes are heterochromatinised in embryogenesis and throughout adulthood. In this species, a non-essential B chromosome can escape paternal genome elimination, thereby enhancing its transmission rate to the next generation. Previous studies show that the B chromosome escapes elimination by changing its chromatin compaction during meiosis to resemble that of maternal chromosomes. Although the exact mechanism underlying this change is poorly understood. Here we investigated histone methylation and acetylation modifications, as well as the Heterochromatin Protein 1 (HP1), to characterise differences between maternal, paternal and B chromosomes during male meiosis of *P. viburni*. Maternal and paternal chromosomes show distinct histone modification patterns, with marks associated with euchromatin present on maternal chromosomes and marks associated with heterochromatin present on paternal chromosomes. We then identified key histone modification changes that coincide with chromatin remodelling of the B chromosome, which allows it to segregate with maternal chromosomes. In addition, we showed that these chromatin modifications occur regardless of the parental origin of the B chromosome. Overall, our findings support the role of histone modifications for proper chromosome segregation during meiosis in mealybugs and provide insight into the mechanisms by which the B chromosome exploits PGE for its preferential transmission.

## Introduction

The faithful segregation of homologous chromosomes during meiosis constitutes the foundation of Mendelian inheritance. However, there are numerous exceptions to this rule, challenging the ubiquity of Mendelian genetics across the tree of life (Burt and Trivers 2006; Ross et al. 2022). One of the most striking examples of non-Mendelian inheritance is meiotic drive (Burt and Trivers 2006; Jaenike 2001; Lindholm et al. 2016) in which specific alleles are transmitted to the offspring at a rate higher than predicted by Mendel’s law. Such “*drivers”* can be single chromosomes, e.g. driving sex chromosomes (Jaenike 2001) or B chromosomes (Ahmad and Martins 2019; Houben et al. 2011) (non-essential chromosomes that are polymorphic in populations) or even involve entire haploid genomes (Herbette and Ross 2023). Under such whole-genome drive, the complete set of chromosomes inherited from one parent is discarded, transmitting only the other parent’s genome. The most widespread type of whole-genome drive is Paternal Genome Elimination (PGE), a genetic system that has evolved repeatedly and is found across tens of thousands of arthropod species (Herbette and Ross 2023). Here males develop from fertilised eggs and are initially diploid, but only maternally derived chromosomes are included in functional sperm. Paternal chromosomes are either lost during early development (embryonic-PGE, found in mites and armoured scale insects) or remain present throughout development and adult life before being eliminated during spermatogenesis (germline-PGE, found in booklice and parasitic lice, most scale insects, sciarid and cecidomyiidae flies, globular springtails and coffee borer beetles) (Herbette and Ross 2023; Ross et al. 2022). In species with germline-PGE the maternal set of genes shows complete drive in male gametogenesis, resembling autosomal driving elements, such as the *t-haplotype* in mice or *Segregation Distorter* in *Drosophila melanogaster* (Zanders and Unckless 2019). These genetic elements distort inheritance patterns to favour their own transmission, eliminating spermatids that lack them and resulting in reduced gamete production (Zanders and Unckless 2019). PGE leads to a similar pattern of spermatid elimination, resulting in the loss of half of all spermatids (Herbette and Ross 2023). However, PGE differs from chromosomal drives in that the drive phenotype is determined by parental origin (paternal vs. maternal), rather than specific nucleotide sequences, and is therefore epigenetically, rather than genetically controlled. There are shared characteristics between PGE species in order to separate chromosomes based on parental origin (Herbette and Ross 2023), such as asymmetric divisions—often associated with monopolar or asymmetric spindles (Courret et al. 2023; Herbette et al. 2021a; Vedanayagam et al. 2023)—and inverted meiotic sequences (in scale insects, mites and probably lice) (Bongiorni et al. 2004; Herbette and Ross 2023; Wrensch et al. 1994). However, while there is clear convergent evolution in terms of modifications to meiotic and mitotic cell division across independent origins of PGE, it remains unclear what cellular and molecular mechanisms underlie the unusual chromosome segregation patterns that lead to paternal genome loss.

Recent work has added considerably to the mechanistic understanding of chromosome-level meiotic drive (Courret et al. 2023; Herbette et al. 2021a; Vedanayagam et al. 2023). Both B chromosomes and chromosomal meiotic drivers increase their transmission either by impairing the viability of competing gametes or by ensuring their own retention during cell division. A common way to achieve this preferential transmission is through manipulating chromatin regulation. For example, Drosophila meiotic drivers like *Segregation Distorter* and Winters *sex-ratio* distorter disrupt chromatin compaction of repetitive sequences during spermiogenesis leading to the elimination of the gamete carrying these sequences (Herbette et al. 2021a; Vedanayagam et al. 2023). Another *sex-ratio* drive system of *D. simulans* involves a dysfunctional allele of HP1D2, a member of the HP1 family, that accumulates on the Y chromosome, disrupting its segregation which results in the production of only X-bearing sperm (Helleu et al. 2016). Similarly, B chromosomes frequently manipulate chromatin regulation to ensure their inclusion in reproductive cells, for example, by interfering with spindle attachment in grasshoppers (Hewitt 1976) or with sister chromatid disjunction (e.g., maize, rye and grass) (Chen et al. 2022). In extreme cases, such as the Paternal Sex Ratio (PSR) chromosomes in *Nasonia vitripennis*, the B chromosome induces the elimination of the entire paternal genome by disrupting histone modifications and chromatin condensation (Aldrich et al. 2017), while PSR itself is resistant to these chromatin modifications. The widespread use of chromatin-based mechanisms in various drive systems is likely due to the fundamental role of chromatin regulation during cell division.

In contrast, the mechanisms underlying whole-genome drive, including PGE, remain largely unknown. In particular, it is unclear how the parental origin of the two chromosome complements are recognised during development and how this affects their segregation and elimination during spermatogenesis. In several PGE clades, maternal and paternal chromosomes in males have striking differences in their chromatin profiles, with in some cases, a strong enrichment of repressive histone marks on paternal chromosomes throughout development and cell type (e.g., in mealybugs, booklice, mites and coffee borer beetles) (Herbette and Ross 2023), while in others, like sciarid flies, differences in the pattern of histone modification are more subtle and cell-specific (Goday and Ruiz 2002; Greciano and Goday 2006). However, it is unclear what role these differences in chromatin profiles play during gametogenesis under PGE. To address this question, we studied the chromatin profile of maternally and paternally derived chromosomes throughout spermatogenesis in the obscure mealybugs (*Pseudococcus viburni*), a small hemipteran insect which is a widespread agricultural pest and reproduces via PGE (Fig. S1A) (Bongiorni et al. 2004; Brown and Nur 1964; Dallai 2012; Nur 1990, 1980). This species is particularly well suited for this work because, in addition to PGE, some individuals also carry a B chromosome (Nur 1962a; Vea et al. 2025). This B chromosome gains a transmission advantage exclusively in males, but not in females, by being included in sperm even when paternally derived (Nur 1969, 1966a, 1966b, 1962a; Vea et al. 2025). Therefore, the B chromosome is able to bypass PGE, which offers an opportunity to study what determines the elimination fate of paternal chromosomes.

In *P. viburni* and related mealybugs, paternally derived chromosomes in males (but not females) undergo heterochromatinisation during early embryonic development, becoming highly compact and transcriptionally silent, while maternal chromosomes remain euchromatic (Fig. S1A) (Bongiorni et al. 2001; Brown and Nelson-Rees 1961; Brown and Nur 1964; de la Filia et al. 2021). This silencing of the paternal genome persists throughout embryogenesis and adulthood, though paternal chromosomes can revert to a euchromatic state in some tissues (e.g., Malpighian tubules, cyst cells) (Nur 1967). Male meiosis in mealybugs is unusual in several ways (Bongiorni et al. 2004) (1) they have holocentric chromosomes, so spindles attach along the lengths of the chromosomes; (2) there is no pairing and crossover between maternal and paternal chromosomes; (3) meiosis follows an inverted sequence compared to canonical meiosis (i.e., sister chromatids separate in meiosis I rather than meiosis II, while homologous chromosomes segregate based on their parental origin during meiosis II) (Bongiorni et al. 2004), which involves a monopolar spindle (Bongiorni et al. 2004; Hughes-Schrader 1948a). This unusual meiosis results in two types of spermatids: those containing euchromatic maternal chromosomes, which mature into functional sperm, while those containing heterochromatic paternal chromosomes are degraded (Hughes-Schrader 1948a) (Fig. S1A). The presence of a B chromosome in *P. viburni* was first discovered by Uzi Nur in the 60s–80s (Nur 1962a; Nur and Brett 1988, 1987, 1985), but has not been studied since then until it was recently rediscovered (Vea et al. 2025). Previous studies suggest that the B chromosome alters its chromatin structure at the onset of meiosis (Nur 1962a; Nur and Brett 1988; Vea et al. 2025). Initially, the B chromosome is heterochromatic regardless of its parental origin and clusters with paternal chromosomes (Nur 1962a). During late prophase I, the B chromosome becomes less compacted, and this allows it to segregate with euchromatic maternal chromosomes during both meiotic divisions, avoiding elimination (Nur 1962a). However, the host genome influences the transmission rate of the B chromosome by affecting its ability to modify chromatin compaction, with drive rates varying from 10 to 95% (Nur 1969, 1966b, 1966a; Nur and Brett 1988, 1985). Based on cytological studies and previous findings that identified overexpression of the B-linked histone acetyltransferase CBP/p300 during male meiosis (Vea et al. 2025), we hypothesise that histone modifications play a key role in determining B chromosome behaviour during meiosis.

In males of the closely related species *Planococcus citri*, paternally and maternally inherited chromosomes show differences in histone modifications enrichment (Bongiorni et al. 2009; Buglia and Ferraro 2004; Ferraro et al. 2001; Kourmouli et al. 2004; Prantera and Bongiorni 2012). HP1 and histone marks associated with heterochromatin and gene silencing, such as H3K9me2/3 and H4K20me3, are enriched on paternal chromosomes during meiosis. However, the specific histone modification patterns in *P. viburni* remain unknown, and the critical marks regulating paternal, maternal, and B chromosome behaviour have yet to be identified. Our study aimed to fill this gap by focusing on meiosis, a critical moment in which 1) the paternal and maternal genomes segregate into functional and non-functional spermatids; 2) the B chromosome gains a transmission advantage. Based on cytology analyses, we generated a detailed catalogue of histone modification patterns on paternal and maternal chromosomes throughout male meiosis, which revealed distinct chromatin profiles for each set. Heterochromatin marks were highly enriched on paternal chromosomes, while histone acetylation was restricted to maternal chromosomes. The B chromosome, initially heterochromatic, gained acetylation marks and adopted an epigenetic identity similar to that of the maternal chromosomes at prometaphase I, without losing its original marks. This shift is independent of the parental origin of the B chromosome and coincided with changes in chromatin compaction and in positioning, allowing the B chromosome to segregate with maternal chromosomes. Our findings show that epigenetic identity and chromatin compaction determine chromosome behaviour during mealybug meiosis, allowing the B chromosome to exploit this mechanism for its preferential transmission.

## Results

### Overview of chromosome behaviours and chromatin dynamics during spermatogenesis in *P. viburni*

The first description of male meiosis in *P. viburni* was published in the 60s–80s (Nur 1962a; Nur and Brett 1988, 1987, 1985), based on light microscopy observations. We redefine these meiotic stages with higher resolution using fluorescence microscopy. Maternal and paternal chromosomes have different levels of chromosome compaction in meiosis, which correlates positively with DNA dye uptake, thus allowing each type of chromosome to be identified based on the intensity of DAPI or Hoechst staining. In addition, during meiosis, the paternal and maternal chromosomes have distinct localisations in the nucleus (Hughes-Schrader 1948b; Nur 1962a; Nur and Brett 1988). Spermatogenesis in mealybugs consists of three consecutive stages: mitosis, meiosis, and spermiogenesis, which coincide with developmental stages (Fig. S1B). More specifically, in *P. viburni*, meiosis occurs during the transition from the second to the third instar larval stage (Nur 1962a; Vea et al. 2025). During the mitotic phase, spermatogonial cells undergo four rounds of division with incomplete cytokinesis, forming a cyst of 16 primary spermatocytes interconnected via cytoplasmic bridges (Buglia and Ferraro 2004). These primary spermatocytes then undergo two meiotic divisions, resulting in 64 haploid spermatids (Fig. S1B). Of these, half contain the paternally derived genome and subsequently degrade, while the remaining half, carrying the maternally derived genome, mature into two sperm bundles, each comprising 16 spermatozoa (Fig. S1B) (Hughes-Schrader 1948b; Nur 1962b).

**Fig. 1:**
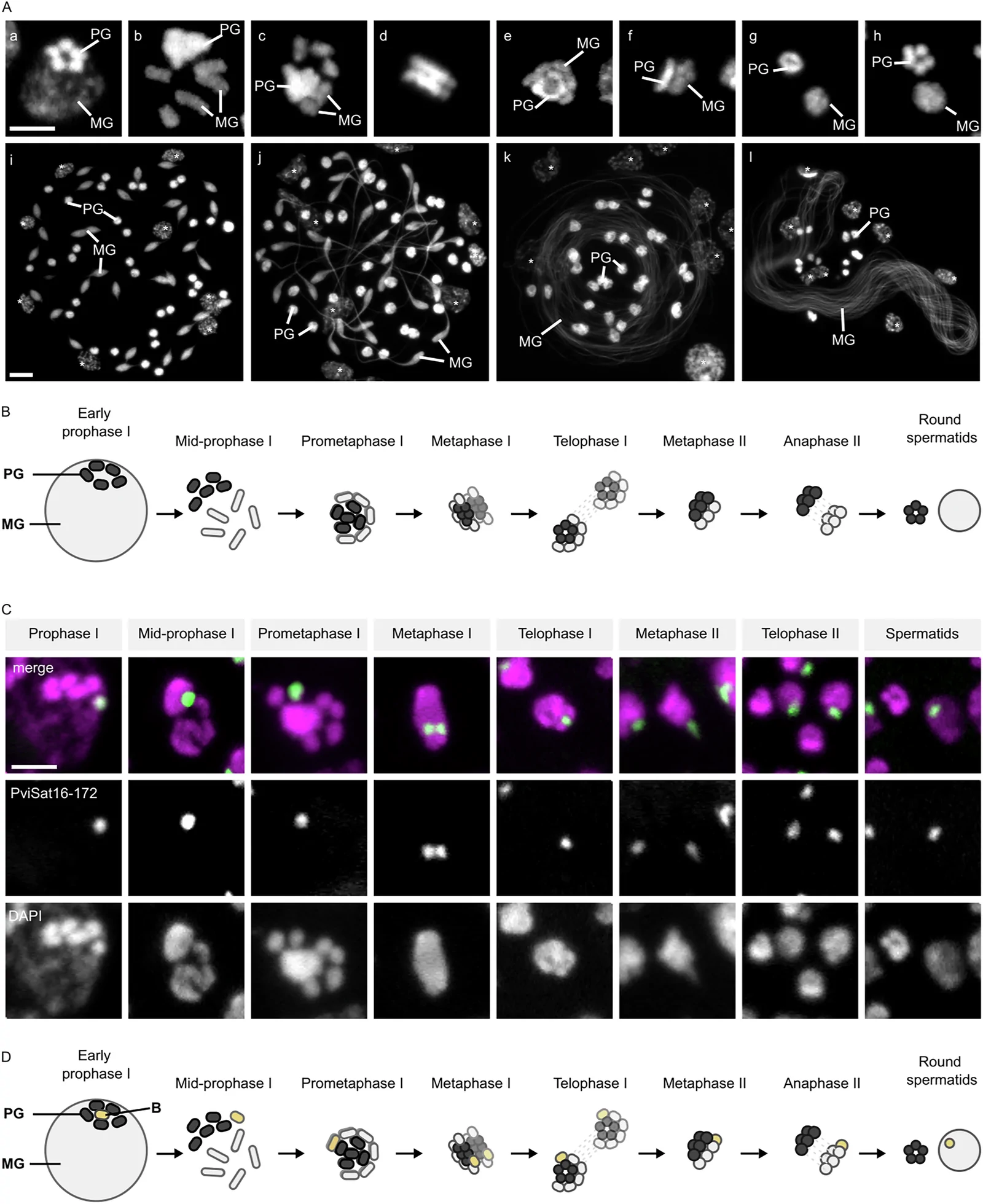
*P. viburni* male meiosis stages in wild-type (2n = 10 chromosomes) and B-carrying (2n = 10 + 1B chromosomes) individuals. **A** Representative images of spermatogenesis stages (Hoechst staining). **a**–**e** First meiotic division (equational). **a** Early prophase I: maternal chromosomes are relaxed, while paternal chromosomes are individualised. **b** Mid-prophase I: maternal chromosomes condense and individualise, paternal chromosomes form a dense cluster. **c** Prometaphase I: paternal chromosomes localise at the centre of the metaphase plate, surrounded by maternal chromosomes. **d** Metaphase I: sister chromatid separation. **e** Telophase I: chromosome arrangement remains the same as in metaphase I. **f**, **g** Second meiotic division (reductional). **f** Metaphase II: homologous chromosomes are distributed on either side of the metaphase plate according to their parental origin. **g** Telophase II: nuclei carrying paternal chromosomes (top left) and maternal chromosomes (bottom right). **h**–**l** Spermiogenesis. **h** Round spermatids with condensed paternal chromosomes. **i** Early elongation: 32 elongating spermatids, 32 round nuclei. **j** Mid-elongation: 32 elongating spermatids, 32 round nuclei. **k** Late elongation: 32 elongated spermatids, 32 round nuclei. **l** Sperm bundle formation and degradation (<32 round nuclei). Asterisks indicate somatic nuclei. **B**, **D** Schematic representation of meiotic divisions and the characteristic position of chromosomes in wild-type males (**B**) or in males carrying one B chromosome (**D**). Light grey: maternal chromosomes; dark grey: paternal chromosomes; yellow: B chromosome. **C** Representative images of meiotic stages in a male carrying one B chromosome, identified by a FISH probe targeting the PviSat16-172 satellite repeats (green); DNA is stained with DAPI (magenta). The B chromosome segregates with paternal chromosomes at the start of meiosis and later joins maternal chromosomes in prometaphase I. Metaphase I shows the equational division of B chromosome sister chromatids. The B chromosome remains associated with maternal chromosomes until the end of meiosis. Scale bars: 5 µm (**A** **a**–**h** and **C**); 10 µm (**A** **i**–**l**). MG Maternal genome, PG Paternal genome, B B chromosome.

We begin by examining spermatogenesis in *P. viburni* males in a non-B carrying line (2n = 10 chromosomes), using Hoechst stain (Fig. 1A, B). Early in prophase of meiosis I, the five paternally derived homologous chromosomes are highly condensed, individualised, and clustered at the nuclear periphery (Fig. 1A-a). In contrast, the five maternal chromosomes remain euchromatic and loosely distributed throughout the nucleus. As prophase progresses, maternal chromosomes condense and individualise, forming an arrangement with the paternal chromosomes tightly clustered at the centre and the less condensed maternal chromosomes distributed around them (Fig. 1A-b and A-c). Homologous chromosomes remain unpaired and do not undergo recombination. This inner-paternal and outer-maternal organisation of chromosomes continues during meiosis I. Sister chromatids are separated at anaphase I (Fig. 1A-d), and two daughter cells are produced in telophase I (Fig. 1A-e). At this stage, both maternal and paternal chromosomes are smaller and more condensed than in prometaphase I, but still retain their differential level of Hoechst intensity. Paternal chromosomes form a dense cluster with a visible “hole” at the centre (Fig. 1A-e), surrounded by the maternal chromosomes. After telophase I, chromosomes directly enter metaphase with homologous chromosomes arranged on either side of the metaphase plate according to their parental origin (maternal vs paternal) (Fig. 1A-f).

In anaphase II, paternal and maternal chromosomes separate, with maternal chromosomes slightly less condensed than paternal chromosomes (Fig. 1A-f) (Nur 1962a). At the end of meiosis, paternal chromosomes retain their characteristic central “hole” that distinguishes them from maternal chromosomes (Fig. 1A-g). Cytokinesis is suppressed at the end of meiosis II and a fusion event occurs between two cells to form a quadrinucleate spermatid (Hughes-Schrader 1948b). After meiosis, the nucleus of functional spermatids decondenses and adopts a round shape (Fig. 1A-h), while paternal chromosomes remain condensed and individualised; later they form a highly compact and small nucleus (Fig. 1A-i). The fate of these spermatid nuclei depends on their chromosomal inheritance: functional spermatids carrying the maternal genome differentiate into mature sperm cells undergoing spermiogenesis, while non-functional spermatids with the paternal genome form aberrant spermatids that eventually degrade (Fig. 1A-i to A-l) (Hughes-Schrader 1948b). During spermiogenesis, functional spermatids undergo extensive chromatin reorganisation. This process is accompanied by elongation of the spermatid nuclei, beginning with a droplet shape to a thread-like shape of approximately 300 µm in length (Nur 1962b) (Fig. 1A-i to A-l). Mature spermatozoa form bundles of around 16 genetically identical sperm cells, completing spermatogenesis (Fig. S1B) (Brown and Nur 1964; Nur 1962b).

Next, to characterise the behaviour of the B chromosome during meiosis, we performed fluorescent in situ hybridisation (FISH) using previously described probes targeting satellite sequences highly enriched on the B chromosome and absent from the autosomes (Vea et al. 2025). In males carrying a B chromosome, we observed that the B chromosome clusters with paternally derived chromosomes prior to meiosis (Fig. 1C, D). However, in prometaphase I, the B chromosome transitions to the outer region of the metaphase plate where the maternal chromosomes are positioned, consistent with earlier observations by Nur (Nur 1962a) (Fig. 1C, D). While Nur observed the B chromosome becoming even less condensed than maternal chromosomes during the transition from prophase I to prometaphase I, we did not see any change in compaction of the B chromosome in the FISH experiment. However, we were able to see this phenomenon in immunostaining experiments (see Figs. 4 and 5), likely because the DNA denaturation required for FISH significantly alters the chromatin structure, making it more difficult to assess these changes. Axial views of the metaphase I plate revealed symmetrical distribution of sister chromatids, as indicated by the two distinct probe signals corresponding to the sister chromatids of the B chromosome (Fig. 1C, D). Following this transition, the B chromosome remains associated with the maternal chromosomes throughout meiosis.

### The histone modifications H3K9me3, H3K27me3, and H4K20me3 mark paternal chromatin during meiosis

One of the most intriguing aspects of PGE in mealybugs is the stark difference in chromatin compaction between paternal and maternal chromosomes in male somatic and meiotic cells. We first analysed the chromatin composition of paternal and maternal chromosomes during male meiosis in a line without B chromosomes.

**Fig. 2:**
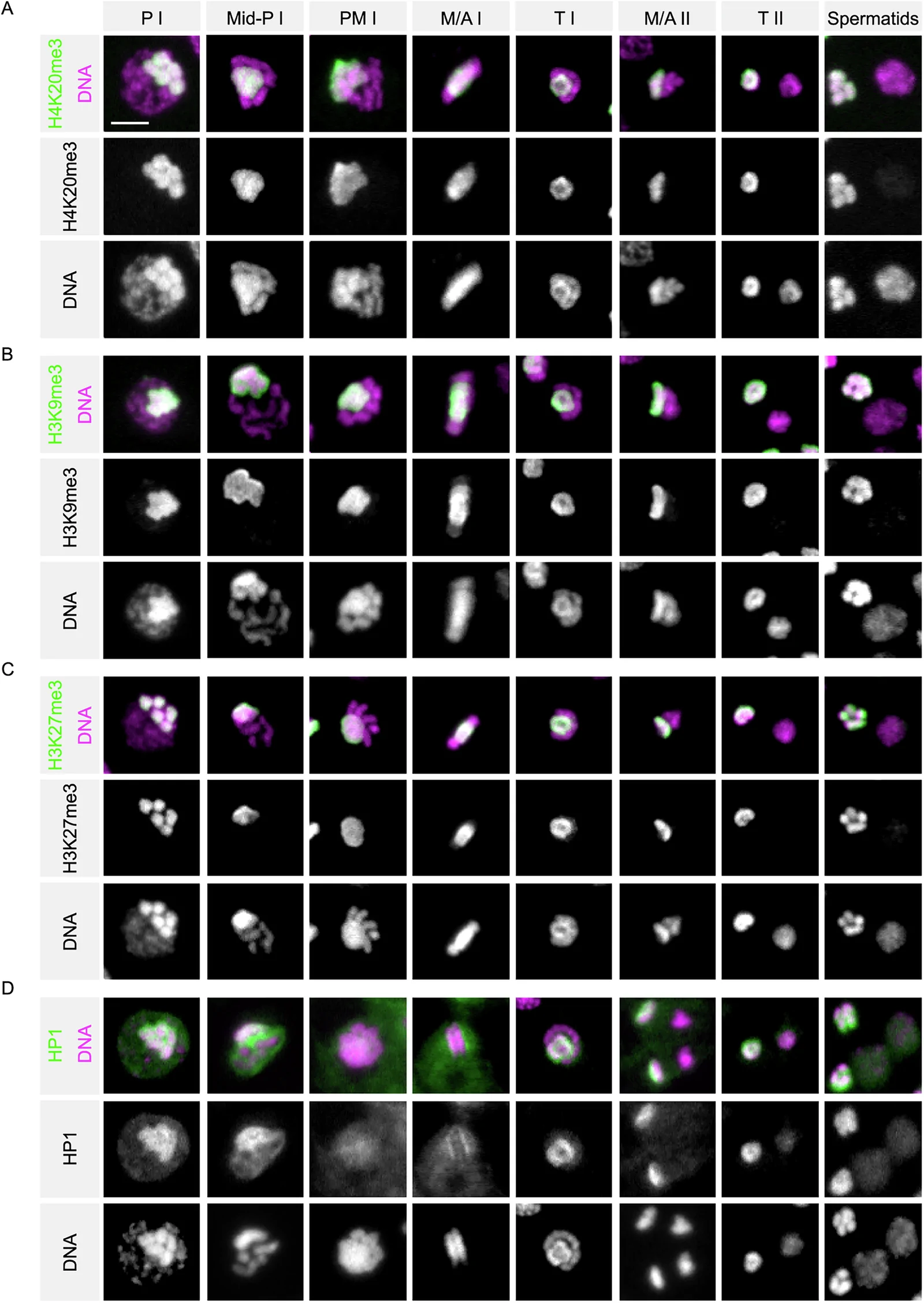
Immunofluorescence images of histone methylation marks and HP1 protein during male meiosis. **A** H4K20me3, (**B**) H3K9me3, (**C**) H3K27me3, (**D**) HP1 in green; DNA (Hoechst) in magenta. Paternal chromosomes are enriched for H4K20me3, H3K9me3, and H3K27me3 throughout meiosis. HP1 is present in the nuclei and is enriched on paternal chromosomes during prophase I and mid-prophase I. In prometaphase, HP1 is absent from chromatin. At metaphase I, HP1 localises to the centromere, forming two distinct lines at the outer edges of the metaphase plate. In telophase I, HP1 reappears on paternal chromosomes and remains on paternal chromosomes during metaphase II. HP1 is detected in both nuclei in telophase II and round spermatids. (P I prophase I, Mid-P I mid-prophase I, PM I prometaphase I, M/A I metaphase/anaphase I, T I telophase I, M/A II metaphase/anaphase II, T II telophase II). Scale bar: 5 µm.

In order to visualise heterochromatin and silent chromatin, we choose to examine three histone modifications which are known to be associated with heterochromatin formation and maintenance in other organisms (Agredo and Kasinski 2023; Dormann et al. 2006; Trojer and Reinberg 2007): the trimethylation of lysine 9 of the histone H3 (H3K9me3), the trimethylation of lysine 27 of the histone H3 (H3K27me3) and the trimethylation of lysine 20 of the histone H4 (H4K20me3). We performed immunostaining experiments using antibodies against these highly conserved histone modifications. We found that all three of these marks showed similar patterns (Fig. 2); they were highly enriched on paternal chromosomes at all stages of meiosis. In contrast, maternal chromosomes displayed background levels of staining for these modifications, likely because small heterochromatin domains are still necessary to ensure telomere functions and silencing of genes and repetitive sequences (Saksouk et al. 2015).

In addition to characterising the distribution of histone marks, we also examined the dynamics of HP1, a major heterochromatin component that binds to chromatin at H3K9me3 sites. HP1 can oligomerise, contributing to the formation and maintenance of heterochromatin (Canzio et al. 2011). We used an antibody targeting *D. melanogaster* HP1 (James and Elgin 1986), previously validated in *P. citri* where it was shown to recognise PCHET2, one ortholog of HP1 (Bongiorni et al. 2001). PCHET2 localises on paternal chromosomes in blastoderm and during spermatogenesis (Bongiorni et al. 2009, 2007, 2001; Buglia and Ferraro 2004). Our observations revealed that HP1 in *P. viburni* localises to meiotic cell nuclei and is particularly enriched on paternal chromosomes during prophase I (Fig. 2D). However, during the following meiotic stages, HP1 is displaced and relocalised to the centromere all along the holocentric chromosomes during prometaphase and metaphase I (Fig. 2D). This relocalisation is also observed in other model organisms (Dormann et al. 2006; Maison and Almouzni 2004). HP1 reappears on paternal chromosomes in telophase I and during meiosis II. At the end of meiosis, HP1 was present in the non-functional spermatid nuclei carrying paternal chromosomes and was also present at low levels in functional spermatids (Fig. 2D).

Overall, our findings demonstrate that paternally derived chromosomes are highly enriched with heterochromatin marks and HP1 throughout meiosis, whereas these marks were present at very low levels on maternally derived chromosomes. This strong enrichment is consistent with the extensive chromatin compaction observed for paternal chromosomes.

### The histone modifications H4K16ac, H3K18ac, and H3K27ac exclusively mark maternal chromosomes during meiosis

After identifying histone marks specific to paternal chromosomes, we next sought to identify those unique to maternal chromosomes. We focused on histone modifications associated with euchromatin, as the maternal genome is euchromatic and is the only copy of the genome expressed in males (de la Filia et al., 2021). We examined by immunostaining H4K16ac (acetylation of lysine 16 on histone H4) associated with actively expressed genes (Shogren-Knaak et al. 2006). Based on our work, which identifies the expression of a truncated version of a B-linked CBP/p300 ortholog during male meiosis (Vea et al. 2025), we examined two acetylation marks catalysed by the CBP/p300 HAT in Drosophila: H3K18ac (acetylation of lysine 18 on histone H3) and H3K27ac (acetylation of lysine 27 on histone H3) (Hundertmark et al. 2018).

**Fig. 3:**
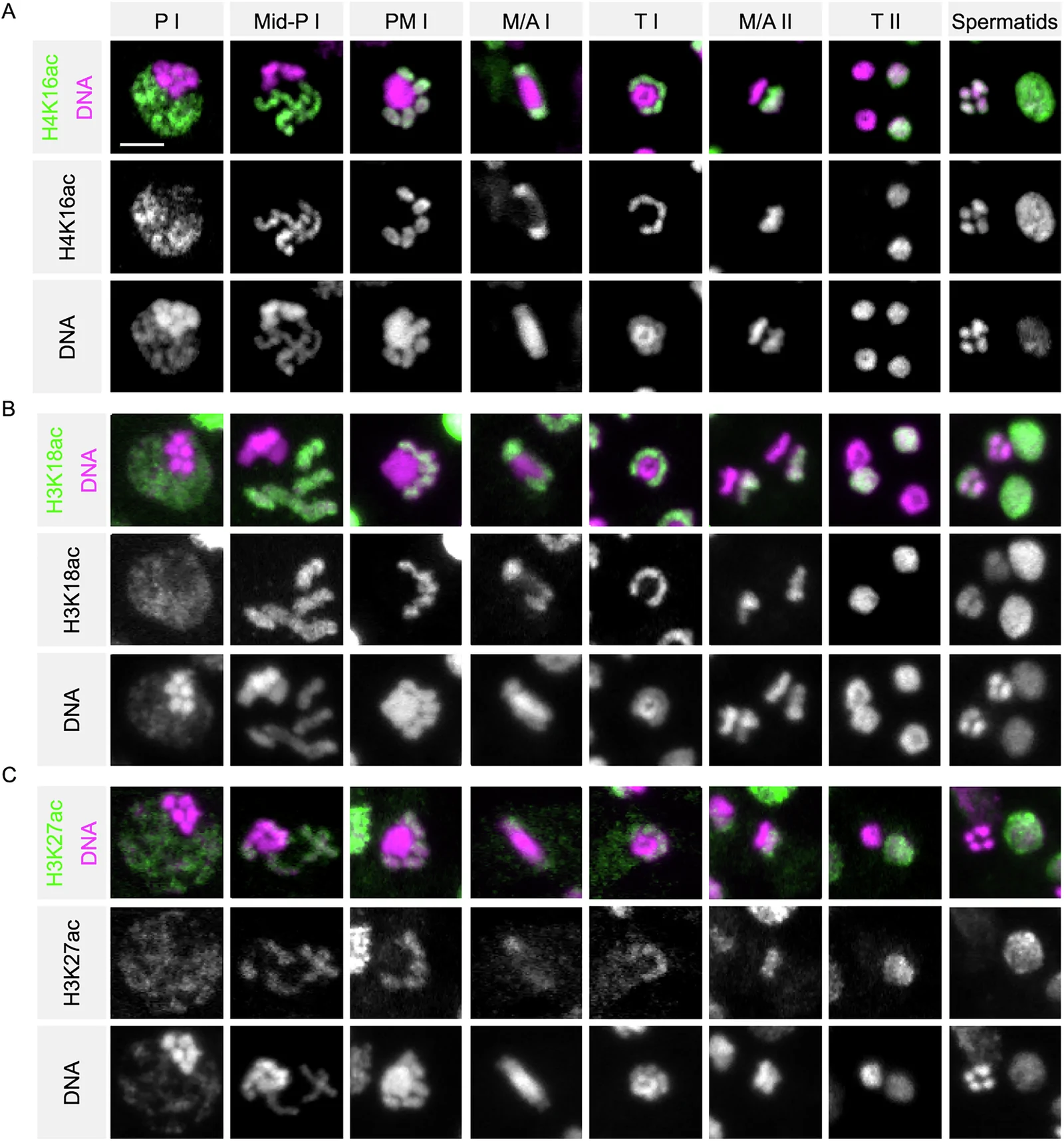
Immunofluorescence images of histone acetylation marks during male meiosis. **A** H4K16ac, (**B**) H3K18ac, (**C**) H3K27ac in green; DNA (Hoechst) in magenta. Maternal chromosomes are enriched for H4K16ac, H3K18ac, and H3K27ac throughout meiosis, while these marks are absent from paternal chromosomes. (P I prophase I, Mid-P I mid-prophase I, PM I prometaphase I, M/A I metaphase/anaphase I, T I telophase I, M/A II metaphase/anaphase II, T II telophase II). Scale bar: 5 µm.

During prophase I, maternal chromosomes were highly enriched for all three acetylation marks (H4K16ac, H3K18ac, and H3K27ac), consistent with transcriptionally active and open chromatin (Fig. 3). In contrast, paternal chromosomes lacked the three acetylation marks consistent with their heterochromatic and silenced state (Fig. 3). These acetylation marks remained exclusively present on maternal chromosomes throughout both meiotic divisions. An exception to this pattern was observed when both types of spermatids (those carrying either the maternal or paternal genome) showed an increase in acetylation levels at the end of meiosis, just before nuclear elongation (Fig. 3). Overall, these findings indicate that histone acetylations are exclusive markers of maternally derived chromosomes during meiosis. After meiosis, acetylation might serve a different role, facilitating the chromatin remodelling required for protamine incorporation and chromatin compaction in functional spermatids.

### Changes in the epigenetic identity of the B chromosome during prophase I of male meiosis

We then investigate how the B chromosome gains a transmission advantage by joining the maternal chromosomes in prophase I. As stated earlier, the B chromosome can change its chromatin compaction during meiosis (Nur 1962a; Nur and Brett 1988; Vea et al. 2025). Although the specific histone modifications responsible for this change in chromatin state remain unknown. Using the same antibodies and cytological approach, we examined the histone modifications present in a B-carrying line (2n = 10 + 1B) to determine: 1) which histone marks are present on the B chromosome and how they change during meiosis, and 2) whether the presence of the B chromosome influences the chromatin composition of the autosomes.

**Fig. 4:**
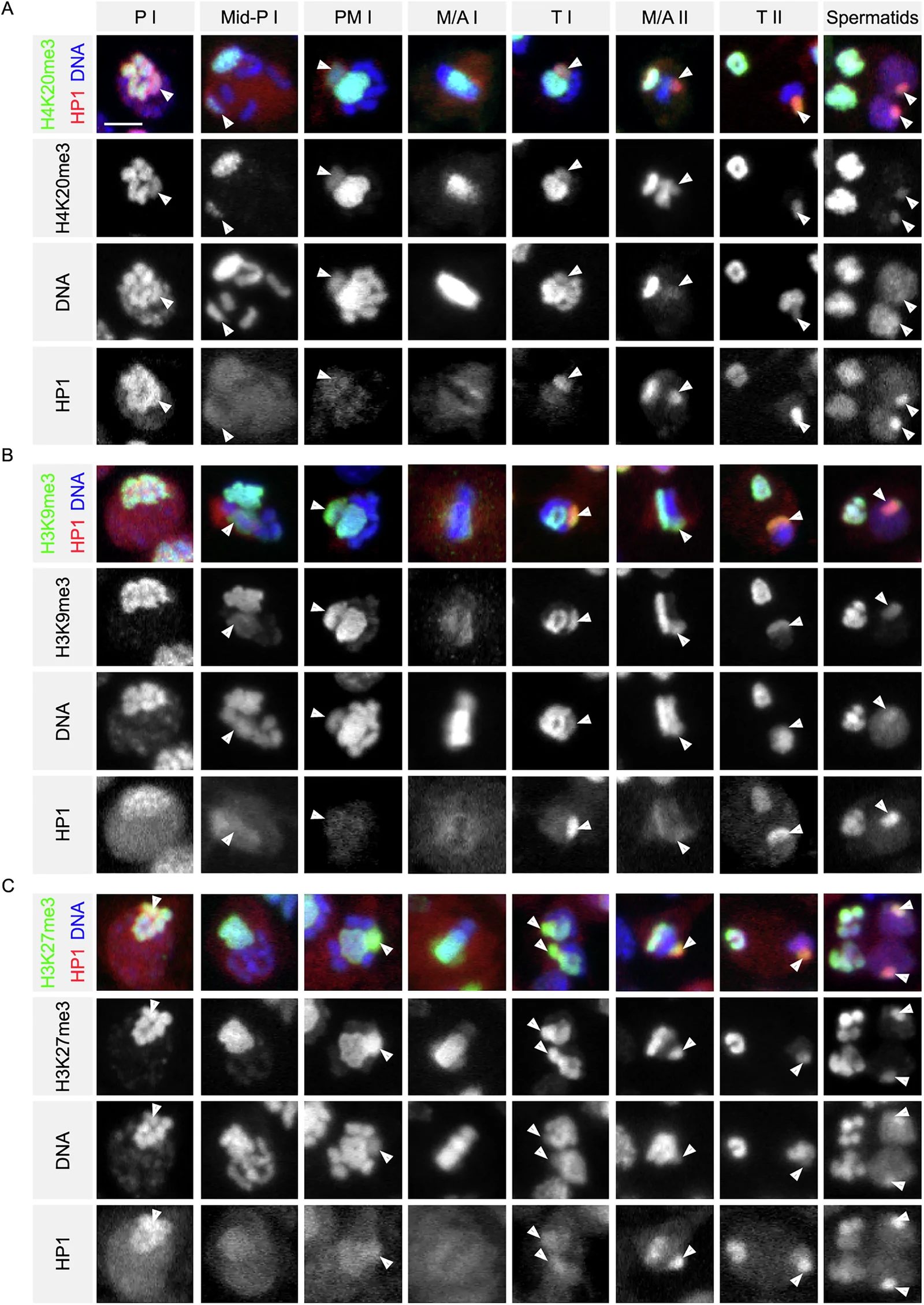
Immunofluorescence images showing the distribution of histone methylation marks and HP1 in a line carrying a B chromosome. **A** H4K20me3, (**B**) H3K9me3, (**C**) H3K27me3 in green; HP1 in red; DNA (Hoechst) in blue. All three marks are present on the B chromosome throughout meiosis even after the B chromosome has undergone changes in chromatin compaction (prometaphase I), which are visible with a less intense Hoechst staining than the other chromosomes. HP1 serves as an additional marker for the B chromosome in addition to the visible change in intensity of Hoechst staining, as HP1 is more enriched on the B chromosome than on the other chromosomes during telophase I and meiosis II. Arrowheads point to B chromosomes when visible. (P I prophase I, Mid-P I mid-prophase I, PM I prometaphase I, M/A I metaphase/anaphase I, T I telophase I, M/A II metaphase/anaphase II, T II telophase II). Scale bar: 5 µm.

**Fig. 5:**
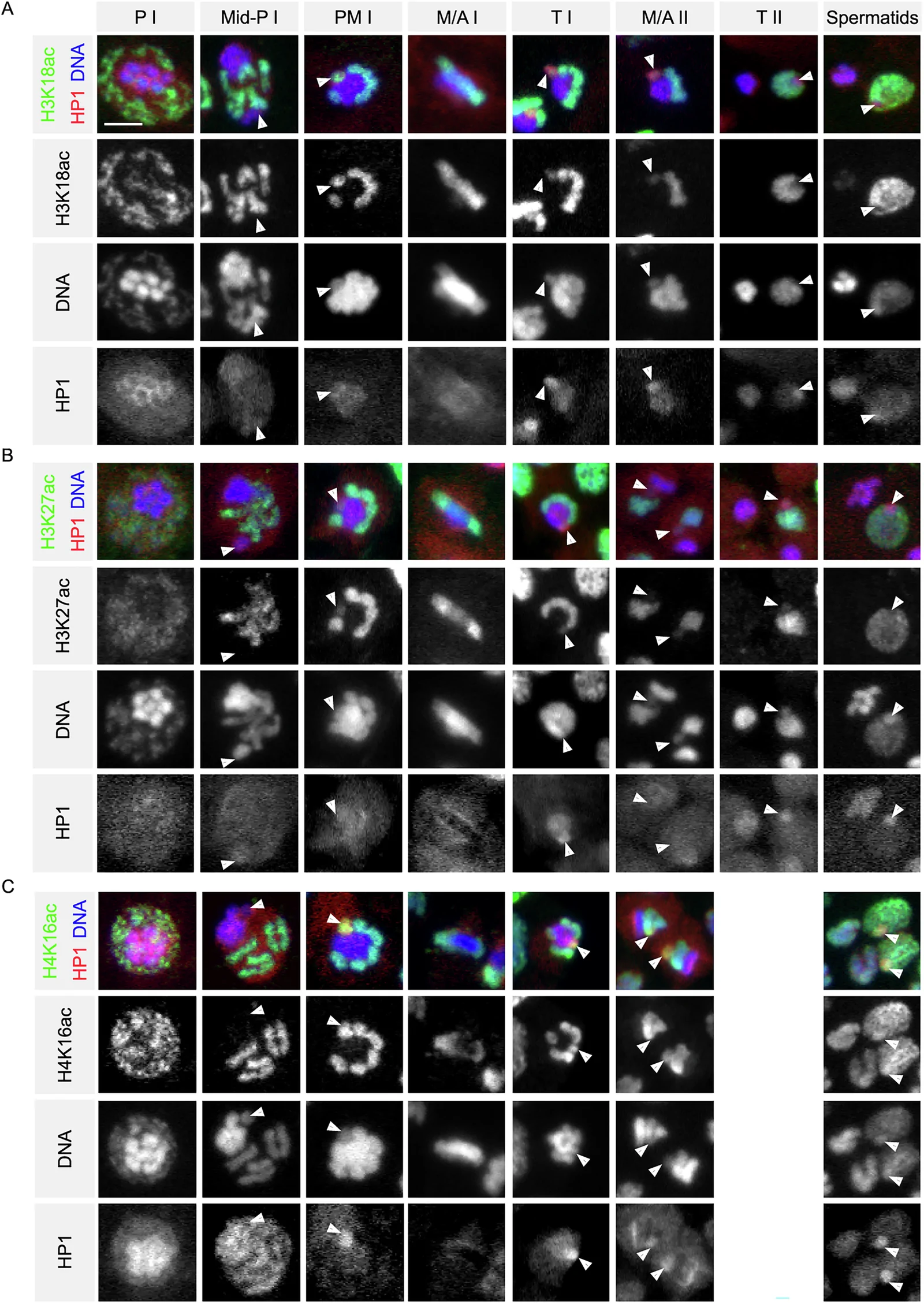
Immunofluorescence images showing the distribution of histone acetylation marks and HP1 in a line carrying a B chromosome. **A** H3K18ac, (**B**) H3K27ac, (**C**) H4K16ac in green; HP1 in red; DNA (Hoechst) in blue. All three marks (H3K18ac, H3K27ac, and H4K16ac) are absent from the B chromosome during prophase I and mid-prophase I (when the B chromosome begins to decondense). In prometaphase I, all three marks appear on the B chromosome as it relocates with the maternal chromosomes and remains present throughout meiosis. HP1 serves as an additional marker for the B chromosome in addition to the visible change in intensity of Hoechst staining. Arrowheads point to B chromosomes when visible. (P I prophase I, Mid-P I mid-prophase I, PM I prometaphase I, M/A I metaphase/anaphase I, T I telophase I, M/A II metaphase/anaphase II, T II telophase II). Scale bar: 5 µm.

We observed no significant changes in the chromatin of paternal and maternal chromosomes in the presence of the B chromosome for all the histone modifications studied and for HP1 (Figs. 4 and 5). Suggesting that the B chromosome does not affect the chromatin of other chromosomes. However, we found clear changes in the chromatin profile of the B chromosome.

During early prophase I, the B chromosome was highly heterochromatic and clusters with the paternal chromosomes (Fig. 4). The B chromosome had the same heterochromatic marks as paternal chromosomes (H3K9me3, H3K27me3 and H4K20me3) (Fig. 4) and maternal-specific marks (H4K16ac, H3K27ac and H3K18ac) (Fig. 5) were absent. However, as meiosis progresses from early prophase to prometaphase, the B chromosome becomes less compact, even less so than that of the maternal chromosomes, as previously observed (Nur 1962a; Nur and Brett 1988) (Figs. 4 and 5, PM I). During this transition, the B chromosome acquires acetylation marks H4K16ac, H3K27ac and H3K18ac, thus resembling the maternal chromatin composition (Fig. 5). Although all the heterochromatin marks were still present on the B chromosome throughout meiosis (Fig. 4). During the following steps of meiosis I and II, the B chromosome maintained both acetylation marks and heterochromatin marks. Interestingly, in mid-prophase I the chromatin decompaction of the B chromosome can be observed prior to the acetylation of H3K27, H3K18 and H4K16 residues (Fig. 5). This indicates that acetylation of histones is not the first trigger for change in chromatin compaction but could be important for further decompaction and stabilisation of chromatin structure. At the end of meiosis, heterochromatin marks and HP1 were still present on the B chromosome and appeared as a more intense dot in the functional spermatid nuclei (Fig. 4). The B chromosome was also acetylated at this stage but at a lower level than maternal chromosomes (Fig. 5). HP1 was even more enriched on the B chromosome compared to paternal chromosomes and was maintained during metaphase II and anaphase II (Figs. 4 and 5).

**Fig. 6:**
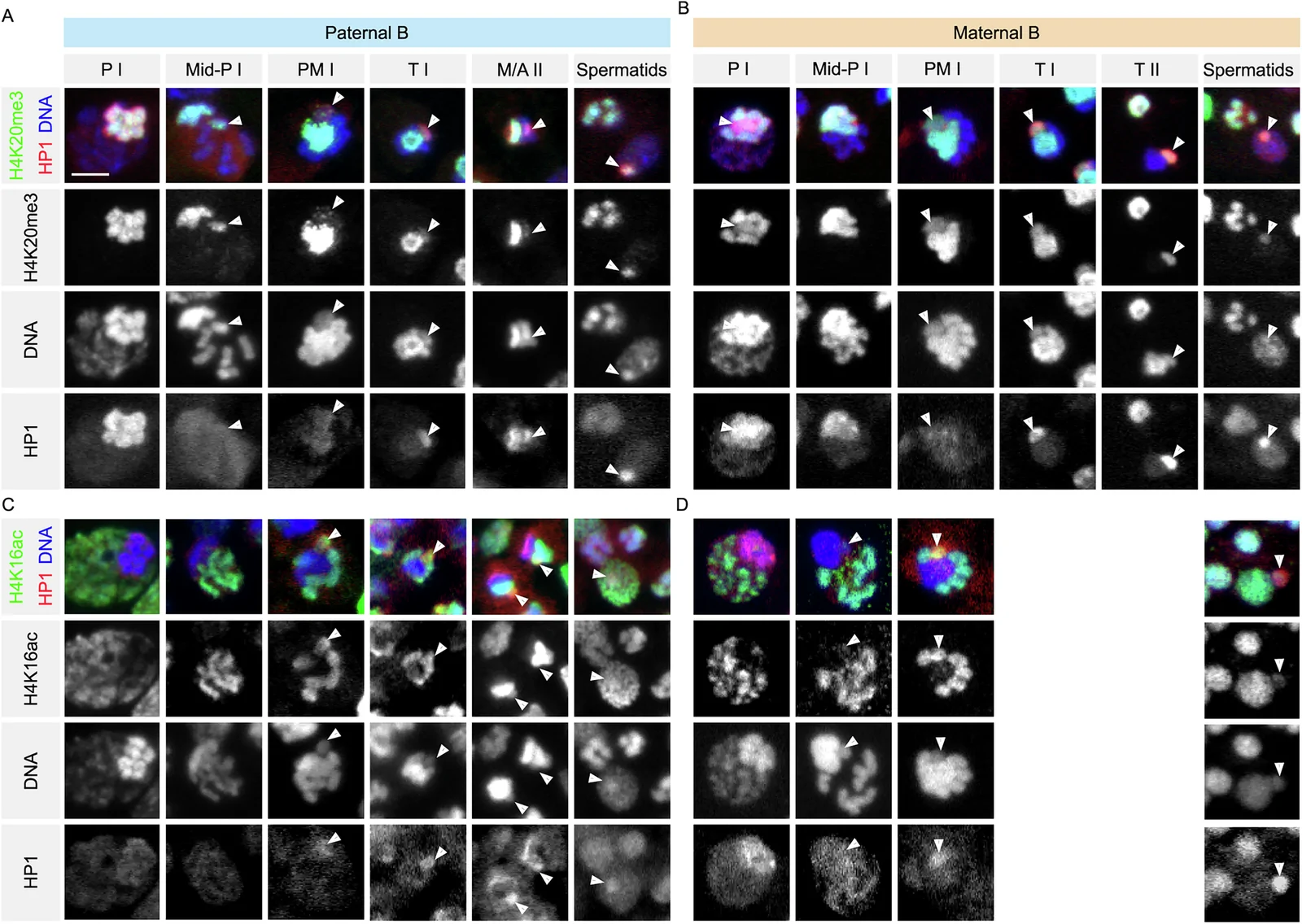
Immunofluorescence images showing the distribution of H4K20me3, H4K16ac, and HP1 during male meiosis when the B chromosome is paternally or maternally inherited. **A**, **B** H4K20me3, (**C**, **D)** H4K16ac in green; HP1 in red; DNA (Hoechst) in blue. **A**, **C** Distribution of H4K20me3, H4K16ac, and HP1 in males that inherited the B chromosome from their father (paternal B). **B**, **D** Distribution of H4K20me3, H4K16ac, and HP1 in males that inherited the B chromosome from their mother (maternal B). Regardless of its parental origin, both histone marks and HP1 show a similar distribution on the B chromosome: H4K20me3 is present on the B chromosome at all stages of meiosis (**A**, **B**), while H4K16ac is absent before prometaphase I, appearing on the B chromosome at this stage and remaining throughout meiosis (**C**, **D**). HP1 serves as an additional marker for the B chromosome in addition to the visible change in intensity of Hoechst staining. Arrowheads point to B chromosomes when visible. (P I prophase I, Mid-P I mid-prophase I, PM I prometaphase I, M/A I metaphase/anaphase I, T I telophase I, M/A II metaphase/anaphase II, T II telophase II). Scale bar: 5 µm.

We also investigated whether paternal or maternal inheritance of the B chromosome has an effect on these switches of chromatin structure and histone modifications. Nur et al. previously observed that the chromatin decompaction of the B chromosome occurs independently of parental origin (Nur and Brett 1988), though this conclusion was based solely on DNA staining. To confirm and extend these findings, we performed reciprocal crosses: males carrying B chromosomes were crossed with virgin females lacking B chromosomes to obtain paternally inherited B chromosomes, while the reciprocal cross produced maternally inherited B chromosomes. We analysed paternal-specific histone modification H4K20me3 as well as HP1 and the maternal-specific histone modification H4K16ac (Fig. 6). H4K20me3 was equally present on the B chromosome in prophase I even when inherited from the maternal side (Fig. 6A, B). The B chromosome acquired the acetylation marks H4K16ac during the transition from prophase I to prometaphase I, regardless of whether it was paternally or maternally inherited (Fig. 6C, D).

Taken together, our findings demonstrate that during meiosis I, the B chromosome changes its epigenetic identity with the acquisition of acetylation marks. This change in identity coincides with the moment when the B chromosome decondenses and changes its localisation in the metaphase plate, moving away from the paternal chromosomes to instead associate with the maternal chromosomes. Furthermore, these changes are independent of the parental origin of the B chromosome, which indicates that a single mechanism allows the B chromosome to transition from a paternal-like to a more maternal-like chromatin state. These results suggest that chromosome segregation in mealybugs is determined by chromatin compaction and epigenetic identity, as changes in histone mark pattern are associated with changes in chromosome behaviour during meiosis.

## Discussion

In this study, we characterised the stages of male meiosis in the obscure mealybug *P. viburni*, a species that displays non-Mendelian chromosome segregation of both paternally derived chromosomes, as well as an accessory B chromosome. We provide a detailed description of histone modifications that defined the chromatin profile of maternal, paternal and B chromosomes throughout meiosis (Table 1). These three distinctive profiles are as follows: (1) H3K27ac, H3K18ac and H4K16ac are exclusive to maternal chromosomes and are absent from paternal chromosomes; (2) HP1 and heterochromatic marks (H3K9me3, H3K27me3 and H4K20me3) are highly enriched on paternal chromosomes during meiosis; in contrast, low levels of these marks are found on maternal chromosomes; (3) B chromosomes show the same chromatin profile as paternal chromosomes prior to meiosis but acquire acetylated marks during prophase of meiosis I (H4K16ac, H3K18ac and H3K27ac); (4) this epigenetic switch on the B chromosome coincides with a change in chromatin compaction and position on the metaphase plate, with the B joining the maternal chromosomes; (5) chromatin modifications on the B chromosome are independent of its parental origin.

**Table 1:** Summary of histone modification levels during meiosis by chromosome type (paternal, maternal, and B chromosomes).

Histone modification	Chromosome type	P I	Mid-P I	PM I	M/A I	T I	M/A II	T II	Spermatids
	PG	+	++	+	+	+	+	+	+
	MG	++	++	++	++	++	++	++	+
	B	-	-	-	-	-	-	-	-
	PG	-	-	-	-	-	-	-	++
	MG	++	++	++	++	++	++	++	++
	B	-	+	+	+	+	+	N.D.	+
	PG	-	-	-	-	-	-	-	++
	MG	++	++	++	++	++	++	++	++
	B	-	+	+	+	+	+	+	+
	PG	-	-	-	-	-	-	-	++
	MG	+	+	+	+	+	+	+	++
	B	-	+	+	+	+	+	+	+
	PG	++	++	++	++	++	++	++	++
	MG	+	-	-	-	-	-	-	+
	B	++	++	++	++	++	++	+	+
	PG	++	++	++	++	++	++	++	++
	MG	+	-	-	-	-	-	-	+
	B	++	++	++	++	++	++	++	++
	PG	++	++	++	++	++	++	++	++
	MG	+	-	-	-	-	-	-	+
	B	++	++	+	+	+	+	+	+
	PG	++	+	-	*	++	++ (*)	++	++
	MG	+	-	-	*	-	-	+	+
	B	+	+	+/−	*	+++	+++	++	++

Histone marks enriched on maternal chromosomes (in red) or paternal chromosomes (in blue) are indicated. The relative enrichment levels of histone marks are represented as follows: - (absent/not detectable), + (present at low level), ++ (enriched), +++ (highly enriched), +/− (variable), N.D. (not determined). (*) HP1 is present at the centromeres. (P I prophase I, Mid-P I mid-prophase I, PM I prometaphase I, M/A I metaphase/anaphase I, T I telophase I, M/A II metaphase/anaphase II, T II telophase II). MG Maternal genome, PG Paternal genome, B B chromosome.

### Histone modifications of parental chromosomes

Our results provide a robust description of the dynamics of histone marks during meiotic divisions in *P. viburni*, which help to better understand the differences between maternal and paternal chromatin and how these differences affect their distinct segregation behaviour. The heterochromatinisation of an entire paternal chromosome set is a unique phenomenon and is exclusively found in species that have evolved PGE and therefore may be fundamental to the cellular mechanisms underlying this system. The establishment of the heterochromatic state in males occurs during early embryogenesis (Bongiorni et al. 2001; Brown and Nur 1964; Nur 1990) and leads to the formation of a heterochromatic body similar to the mammalian Barr body (Nur 1990). The difference in chromatin organisation between maternal and paternal chromosomes is maintained throughout both meiotic divisions and persists until the onset of spermatid elongation (Fig. 1A, B) (Hughes-Schrader 1948b). This compact heterochromatic state of paternal chromosomes is characterised by the accumulation of three heterochromatic marks, H3K9me3, H4K20me3 and H3K27me3, alongside the enrichment of HP1, a major component of heterochromatin formation and maintenance that binds to H3K9me3 (Canzio et al. 2014; Dormann et al. 2006) (Fig. 2). This global heterochromatinisation, particularly the accumulation of H3K27me3, is consistent with the repression of paternal genes observed in *P. citri* (de la Filia et al. 2021). These histone modifications remained stable throughout meiosis, with the exception of HP1, whose dynamic is discussed later. H3K9me3 and H4K20me3 are linked to gene repression and serve as hallmarks of constitutive heterochromatin, usually found in genomic regions rich in repetitive sequences, including telomeres, centromeres, transposable elements and satellite repeats (Saksouk et al. 2015). Moreover, H3K9me3 and HP1 facilitate the deposition of H4K20me3 (Agredo and Kasinski 2023). Thus, these two marks are often co-localised within the same chromatin region (Agredo and Kasinski 2023). In contrast, H3K27me3 is primarily associated with facultative heterochromatin, a form of heterochromatin found on repressed genes and varying according to cell type (Trojer and Reinberg 2007). Interestingly, the inactive X chromosome in mammalian cells is also marked by all three modifications and HP1 (Chadwick and Willard 2004; Vaskova et al. 2014). However, in mammalian inactive X, H3K27me3 displays an alternating pattern relative to the other two marks, reflecting two distinct mechanisms of heterochromatin formation (Chadwick and Willard 2004; Vaskova et al. 2014). In *P. viburni* we did not observe such a pattern, either because of insufficient resolution or because all three marks occupy the same chromatin regions.

Histone acetylation on lysine residues is generally associated with open chromatin and transcriptionally active genes (Bannister and Kouzarides 2011; Shogren-Knaak et al. 2006). Consistent with this, all three histone acetylations studied (H318ac, H3K27ac and H4K16ac) were restricted to maternal chromosomes in *P. viburni* (Fig. 3). During cell division, a global reduction in histone acetylation is generally observed to ensure proper chromatin condensation and accurate segregation of chromosomes (Hennig and Weyrich 2013; Kruhlak et al. 2001). In *P. viburni*, acetylation levels remain comparable throughout meiosis on maternal chromosomes, suggesting that maintaining these marks during cell divisions might be important for maintaining the epigenetic identity of maternal chromosomes, even though it can interfere with chromatin condensation. Interestingly, all three acetylated marks are absent from paternal chromosomes and thus resemble again what is observed for X chromosome inactivation in mammalian cells (Khalil and Driscoll 2007). This absence of acetylated histones is also consistent with paternal genome silencing observed in mealybugs (de la Filia et al. 2021).

### Non-Mendelian inheritance of maternal and paternal chromosomes through spermatogenesis

In most organisms, homologous chromosome pairing is essential for accurate reductional segregation during meiosis, preventing aneuploidy. However, *P. viburni* and other mealybugs with germline-PGE deviate from this paradigm by lacking homologous chromosome pairing and physically segregating chromosomes based on parental origin. Imprinting mechanisms in *P. viburni* may compensate for the absence of pairing by distinguishing homologous chromosomes, ensuring proper segregation and preventing aneuploidy, though at the cost of independent assortment. Supporting this idea, we observed a striking and stable difference in the chromatin profiles of the maternal and paternal chromosome sets throughout meiotic divisions, ensuring correct homologous chromosome recognition (Figs. 2 and 3).

The holocentric nature of *P. viburni* chromosomes may also play a role, as chromatin state likely has a greater influence on chromosome dynamics compared to monocentric chromosomes such as microtubule interactions or centromeric activity. Therefore, chromatin differences between maternal and paternal sets may influence their behaviour. This also might explain the characteristic metaphase arrangement, with paternal chromosomes localised internally and maternal chromosomes externally (Fig. 1A, B). HP1 dynamics during meiosis further support this model. Usually, HP1 dissociates from chromatin during cell division, except at centromeres, to facilitate condensin loading (Dormann et al. 2006). In *P. viburni*, HP1 is released during the prophase I to metaphase I transition and relocates to centromeric regions, which cluster at the metaphase plate edges in holocentric chromosomes (Heckmann et al. 2014; Marques and Pedrosa-Harand 2016) (Fig. 2D). Additionally, during meiosis II, HP1 localises exclusively to paternal chromosomes, likely due to higher H3K9me3 enrichment (Fig. 2B, D). These observations suggest distinct centromeric activity between maternal and paternal chromosomes, influenced by the differential chromatin state of the homologous chromosomes. This in turn could influence asymmetric distribution of parental chromosomes during meiosis (and consequently the loss of paternal chromosomes).

### Comparisons with previous studies

Previous studies on the closely related species *P. citri* first described the pattern of several histone modifications associated with heterochromatin or euchromatin during embryogenesis (Bongiorni et al. 2007, 2001; Ferraro et al. 2001; Kourmouli et al. 2004), oogenesis (Bongiorni et al. 2004) and spermatogenesis (Bongiorni et al. 2009, 2004; Buglia and Ferraro 2004). In the latter study, similar to our findings, H3K9me3 and PCHET2/HP1 were enriched on paternal chromosomes in pre-meiotic cells (spermatogonia) and quadrinucleate spermatids. However, contrary to our results, H4K20me3 was reported enriched on maternal chromosomes in spermatogonia (Bongiorni et al. 2009). Additionally, H3K9me3, unmodified histone H3 and PCHET2/HP1 were found in the functional nuclei of quadrinucleate spermatids, while H4K20me3 was also in the cytoplasm of these same cells. Although we observed low levels of heterochromatic marks (H3K9me3, H4K20me3 and H3K27me3) and HP1 in the nuclei of functional spermatids, these marks were only slightly enriched beyond background level (Fig. 2). They likely reflect facultative and constitutive heterochromatin that was already present before meiosis and plays an essential role in normal gene expression and chromatin regulation (Maison and Almouzni 2004; Saksouk et al. 2015). We believe that the discrepancies between our findings and previous studies are primarily due to technical artefacts introduced by acid fixatives used in those earlier studies. Acetic acid is known to extract histones from tissues by disrupting their electrostatic affinity for DNA (Dick and Johns 1968; Johansen et al. 2009; Shechter et al. 2007), potentially introducing bias in histone localisation. We confirmed that acid fixative can cause histone to diffuse outside the nucleus by directly comparing acid-based and acid-free fixation protocols (Fig. S2). Therefore, our interpretation is that histones released by acid treatment may have diffused throughout the quadrinucleate spermatids, leading to misinterpretation of their true localisation. Similarly, the association of some histone marks to maternal chromosomes at the end of meiosis or in spermatogonia could also come from the same technical artefact and wrong interpretation, as these observations only occur when cytoplasmic histones are also observed.

### Mechanisms of B chromosome drive

A key focus of this manuscript was to understand the drive mechanism of the B chromosome in *P. viburni* and use these insights to better understand the mechanisms of chromosome elimination during male meiosis in mealybugs. B chromosomes are found in thousands of species across eukaryotes (D’Ambrosio et al. 2017). Unlike essential chromosomes, B chromosomes are not part of the standard chromosome set in a species and can be lost without affecting the organism’s viability (Camacho et al. 2000; Jones et al. 2008). Despite being dispensable, B chromosomes persist through various non-Mendelian strategies that operate before, during, or after meiosis, resulting in a transmission rate higher than 0.5. A number of classic studies led by Uzi Nur suggested that the B relies on changing its level of condensation during meiosis to resemble maternal, rather than paternal chromosomes and that this allows it to segregate into maturing spermatids (Nur 1962a). Here we find support for this hypothesis and show that the B chromosome undergoes profound changes in its chromatin profile to more closely resemble the maternal chromosomes, and this coincides with a change in the B chromosome’s localisation, clustering with the maternal rather than paternal chromosomes (Figs. 4 and 5). We also confirm Nur’s earlier findings that the B chromosome is able to do so independently of its own parental origin (Fig. 6) and therefore seems to have evolved a mechanism to avoid the imprinting mechanism acting on the autosomes (Nur and Brett 1988). Interestingly, the B chromosome in *P. viburni* is not the only B chromosome known to drive through manipulating chromatin regulation. The PSR chromosome found in *N. vitripennis* manipulates their haplodiploid genetic system—where females are diploid and develop from fertilised eggs, while males are haploid and develop from unfertilised eggs—to enhance its transmission (Nur et al. 1988). PSR converts embryos destined to become diploid females into haploid males by making sperm chromatin non-functional (Aldrich et al. 2017; Swim et al. 2012). This disrupted chromatin is eventually lost during early development, resulting in haploid eggs that develop into males (Nur et al. 1988). Consequently, PSR is inherited exclusively through males. The gene *haplodizer* on the PSR chromosome acts as a toxin that induces sperm chromatin disruption, although the exact mechanism remains unclear (Dalla Benetta et al. 2020). Interestingly, this process is associated with abnormal histone mark distribution (i.e., H3K9me3 and H4K20me1) on the affected chromatin (Aldrich et al. 2017). The PSR chromosome itself remains unaffected by these chromatin alterations despite being in the same nucleus, ensuring its proper individualisation, segregation, and persistence. Unlike PSR, the *P. viburni* B chromosome is only able to modify its own chromatin and is unable to modify other chromosomes, even when translocated together with an autosome (Nur and Brett 1988). Nonetheless, the presence of these two chromosomes during spermatogenesis for *P. viburni* or during the first embryonic division for *N. vitripennis*, has the ability to greatly modify chromatin.

One of the key questions in understanding PGE is how cells determine the physical position of chromosomes based on their parental origin and how this eventually leads to their distinct fate. Our results show that histone modifications differ between maternal and paternal chromosomes; however, their role in chromosome positioning remains unclear. A distinctive feature of meiosis in *P. viburni* is the complete absence of pairing and recombination between maternal and paternal chromosomes, accompanied by a highly stereotyped spatial organisation: paternal chromosomes form a central ring surrounded by maternal chromosomes in metaphase I (Hughes-Schrader 1948b; Nur 1962a). This spatial arrangement is thought to be driven by differences in chromatin compaction (Nur 1962a; Nur and Brett 1988). The B chromosome—which in this context serves as a natural mutant—can shift its relative position from the paternal to the maternal chromosome side during the transition from prophase I to metaphase I (Fig. 1C, D) (Nur 1962a). This shift in position is accompanied by a change in chromatin compaction (Figs. 4 and 5). Before meiosis, the B chromosome is fully heterochromatic and consistently associated with paternal chromosomes, regardless of its parental origin (Fig. 6) (Nur and Brett 1988), and carries the same heterochromatin marks (Fig. 4). However, during metaphase I, when the B chromosome decondenses to a state similar to that of maternal chromosomes, it relocalises to instead associate with the maternal chromosomes (Figs. 4 and 5). Further evidence supporting the role of chromatin compaction in chromosome positioning comes from variations in B chromosome behaviour in different genetic backgrounds (Nur and Brett 1988). In some genetic backgrounds, B chromosomes that fail to decompact remain at the centre of the metaphase plate with the paternal chromosomes throughout meiosis. In contrast, B chromosomes that are able to become less compacted successfully relocalise to the periphery of the metaphase plate and segregate alongside maternal chromosomes (Nur and Brett 1988). Supporting these results, we found that B chromosome decompaction is immediately followed by the acquisition of acetylation on multiple lysine residues (H4K16, H3K27, and H3K18), making the B chromosome chromatin profile similar to that of maternal chromosomes (Fig. 5). These acetylations are involved in chromatin relaxation (Kumar et al. 2021) and may facilitate the changes necessary for the B chromosome’s localisation switch during meiosis.

Consistent with H3K18ac and H3K27ac acquisition by the B chromosome during metaphase I (Fig. 5), our previous study identified a B-linked gene, g13953 (Vea et al. 2025), expressed in males when meiosis occurs. This gene encodes a protein containing a conserved histone acetyltransferase (HAT) domain from the CBP/p300 family. Members of this family are conserved HATs that acetylate several lysine residues, including H3K56, H3K18 and H3K27 (Bannister and Kouzarides 2011; Das et al. 2009; Hundertmark et al. 2018). This finding supports a model where the B chromosome expresses this HAT, which is recruited—via an unknown mechanism—to acetylate H3K18 and H3K27. A full-length ortholog of CBP/p300 (g14652) exists on the autosomes, along with another truncated gene (g11059) that shares 56% similarity with the B copy and also contains only the catalytic HAT domain. One hypothesis is that this truncated B-linked protein may interfere with the localisation or function of the full-length autosomal protein, potentially restricting its activity to the B chromosome. g13953 lacks several domains essential for forming multi-protein complexes, which are usually required for histone modifiers to function properly (Brown et al. 2000; Yang and Seto 2007). Whether g13953 is directly responsible for depositing these specific histone marks or encodes a fully functional HAT remains unclear. Further, functional analyses will be needed to elucidate the roles of both the autosomal gene and the B-linked copy in histone acetylation and the chromatin structural changes observed in the B chromosome.

Beside the B-linked CBP/p300 gene, additional complexes likely contribute to chromatin decompaction. Genetic background has been shown to influence B chromosome drive by modulating its chromatin remodelling capacity (Nur and Brett 1988), suggesting the presence of autosomal modifier elements that regulate chromatin structure. The MOF/MYST histone acetyltransferase complex represents a strong candidate; this highly conserved complex acetylates H4K16 (Akhtar and Becker 2001; Hilfiker et al. 1997; Shogren-Knaak et al. 2006; Smith et al. 2005), a modification acquired by the B chromosome alongside H3K27ac and H3K18ac (Fig. 5). Additionally, our observations indicate that decompaction precedes the acquisition of these acetylation marks (Fig. 5, mid-prophase), suggesting these modifications may not initiate decompaction but rather stabilise the newly established chromatin state. Other chromatin remodelers or organisers may participate in this initial decompaction process, including ATP-dependent chromatin remodelers, eviction or replacement of histones, topoisomerase TopII and Condensin I and II complexes. Partial reduction in condensin or TopII binding to DNA could directly contribute to reduced chromatin compaction (Green et al. 2012; Piskadlo et al. 2017; Piskadlo and Oliveira 2017). While the precise mechanism remains unclear, it points toward a complex, multi-step process that is implicated in the chromatin reorganisation of the B chromosome that needs further investigation. Additionally, release of heterochromatin could also achieve decompaction. However, H4K20me3, H3K27me3, and H3K9me3 remain present on the B chromosome (Fig. 4). Surprisingly, we also observed the presence of HP1 on the B chromosome’s chromatin during meiosis I, and with even more consistent retention during meiosis II (Fig. 4). This retention is unexpected, as it goes against the dissociation of HP1 from chromatin during cell division and against the known role of HP1 in chromatin compaction. This is also in opposition with the observed chromatin decompaction of the B chromosome. Such a contradiction could be explained by the functional diversification of one of the ten HP1 orthologs identified in the genome of *P. viburni* (Vea et al. 2025). In mammals, loss of HP1α results in hyper-compaction of chromatin, suggesting a role in the maintenance of a more open chromatin (Bosch-Presegué et al. 2017). In Drosophila, HP1a can bind to gene promoters independently of H3K9me3, which indicates that HP1 may evolve diverse functions outside of heterochromatin formation and maintenance (Figueiredo et al. 2012).

In addition to its gene content, the structural properties of the B chromosome, particularly its repeat content, likely play an important role in driving its transmission. B chromosomes are usually enriched in repetitive elements, including transposable elements or satellite repeats, with often little homology between autosome-repeats and B-repeats (Camacho et al. 2000; Dalla Benetta et al. 2020; Li et al. 2017). In *P. viburni* satellite repeats account for 62.1% of the B chromosome, including several B-specific satellites (Fig. 1C) (Vea et al. 2025). Interestingly, a common mechanism in multiple drive systems involved satellite repeats, and in some cases abnormal chromatin structure at these repeats (Clark and Akera 2021; Herbette et al. 2021b; Hiatt et al. 2002; Muirhead and Presgraves 2021). For instance, in the SD meiotic drive system in *D. melanogaster*, the *Responder* satellite fails to fully incorporate protamines during spermatid maturation, leading to chromatin defects and ultimately to the elimination of spermatids that carry a large number of those repeats (Herbette et al. 2021b). Therefore, B-specific repeats likely play a crucial role in chromatin regulation, possibly by recruiting key chromatin remodelers. Supporting this, when B-autosome translocations are paternally inherited, only the B-derived segments decompact and reposition alongside maternal chromosomes during prophase I, while the autosomal portions remain heterochromatic and internally positioned. This indicates B-specific regions can undergo autonomous chromatin changes without affecting adjacent non-B regions.

## Conclusion

**Fig. 7:**
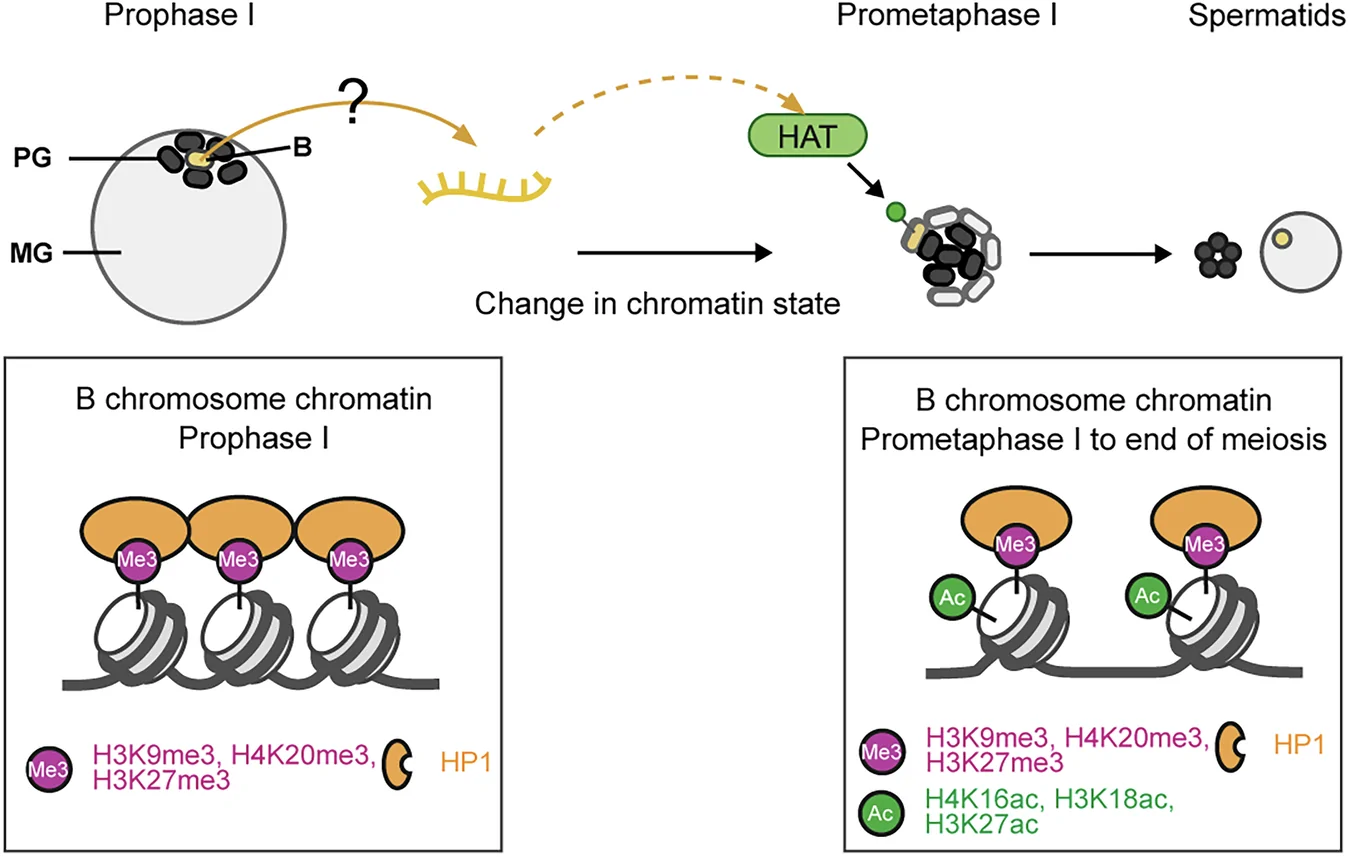
Model for maternal and B chromosome drive. The B chromosome expresses transcripts that modify its chromatin state by promoting acetylation through the activation of histone acetyltransferase (HAT) proteins. This chromatin modification (see boxes) enables the B chromosome to associate with the maternal chromosomes at the periphery of the metaphase plate. The altered chromatin state remains stable until the end of meiosis, ensuring that the B chromosome segregates with the maternal chromosomes and is transmitted to the functional spermatids. MG Maternal genome, PG Paternal genome, B B chromosome.

Taken together, this result suggests that the change in B chromosome behaviour depends on its compaction state and epigenetic identity, which likely also underlies the differential behaviour of maternal and paternal chromosome sets during meiosis. Based on our result and previous works, we can build a hypothetical model for the B-drive mechanism (Fig. 7). The B chromosome achieves its drive mechanism by expressing transcripts encoding proteins such as the CBP/p300-like protein able to acetylate histones or encoding a protein that recruits or activates remodelers and HATs. In addition, B-specific satellite repeats or unique heterochromatin compositions may facilitate the targeted recruitment of these chromatin modifiers exclusively to the B chromosome. This recruitment leads to a remodelling of the chromatin of the B chromosome, making it more closely resemble the maternal chromosomes in terms of histone modifications and chromatin compaction. This resemblance allows the B chromosome to join the maternal chromosomes during meiosis I by an unknown mechanism. Consequently, the B chromosome segregates preferentially with the maternal chromosomes during both meiosis I and II. Ultimately, the B chromosome is inherited in the functional spermatid at a high frequency, driving its preferential transmission across generations. Although the precise cause of the physical separation between the two chromosome sets remains unclear, one possible explanation for the strong influence of chromatin state on their behaviour is the holocentric nature of these chromosomes. Unlike monocentric chromosomes, which have a single localised centromere, holocentric chromosomes possess spindle attachment sites distributed along their entire length. Consequently, changes in chromatin condensation or epigenetic modifications are more likely to affect their movement and segregation during cell division through microtubules and spindle attachment. Future study may help understand the relationship between differences in chromatin state, regulation of spindle attachment and chromosome movement. So far, the *P. viburni* B chromosome is the only B chromosome studied at the cell-phenotype level in a species with PGE. In this system, it exploits the existing asymmetry between the epigenetic identities of chromosomes based on their parental origin. Since PGE is found across thousands of species, it would be interesting to explore whether B chromosomes present in other PGE species similarly exploit this asymmetry and use comparable mechanisms to escape PGE.

## Materials and methods

### Mealybug populations and cultures

We collected egg-laying females in the Royal Botanical Garden in Edinburgh, UK, to establish isofemale lines of *Pseudoccocus viburni*. We used the same rearing method as previously described (Vea et al. 2025). Mealybugs were reared on sprouted potatoes as food and kept in Tupperware boxes with holes cut in the lid, covered with nylon mesh to allow air circulation and lined with paper towels to absorb excess moisture. Cultures were maintained at 25°C with no humidity control and with a 12/12 h day/night photoperiod.

### Hoechst staining and immunofluorescence on squashed testes

The immunostaining protocol was adapted from Grosmaire et al. 2019. First, homemade poly-L-lysine slides were prepared on the same day as dissection as follows: 2 mg/ml poly-L-lysine (Merck Cat#P5899) was applied to the slides and spread using a rubber spatula (Deutsch and Neumann, Cat#2260002). The slides were then heated to 100°C for 5 min. This process was repeated twice. 10 to 20 males of interest stage were collected: 2nd–3rd instars for meiotic cells to 4th instars for mature spermatozoa. Testes were dissected out in DPBS-T (1X DPBS (Merck, Cat#D1408), 0.05% Triton (Merck, Cat#X100)) on slides coated with poly-L-lysine. After removal of excess liquid, testes were fixed by adding 30 µl of 3% paraformaldehyde (Thermo Scientific, Cat#10751395) diluted in 1X DPBS for 5 min at room temperature. A coverslip was placed on top of the sample and then squashed between folded paper towels. Slides were placed in liquid nitrogen until boiling stopped, then the coverslip was quickly removed, and the slides were placed in 100% MeOH (Scientific Laboratory Supplies, Cat#CHE2534) for 1 min in a Coplin staining jar. After three 10 min washes in DPBS-T, the slides were incubated with 30 µl of primary antibodies diluted in DPBS-T in a humid chamber at 4°C overnight. After three washes of 10 min each in DPBS-T, the slides were incubated with secondary antibodies and 0.5 µg/mL of Hoechst (Orbyt, Cat#orb363806) diluted in DPBS-T for 2 h at room temperature. The slides were then washed three times for 10 min each in DPBS-T and mounted in mounting medium (80% glycerol (Merck, Cat#G-5516), 1X DPBS, 0.2% propyl gallate (Merck, Cat#P3130)). For each immunostaining experiment, at least two independent crosses and immunostainings were done.

All antibodies were diluted in DPBS-T, and are as follows:

Primary antibodies: HP1, 1:100 (DHSB by Wallrath, L.L. (James and Elgin 1986) Cat# C1A9; RRID: AB_528276); H4K16ac, 1:100 (Active Motif Cat# 39929; RRID: AB_2753164); H3K9me3, 1:100 (Millipore Cat# 07-523; RRID: AB_310687); H3K9me3, 1:100 (Biovision Cat#6873-25); H3K27me3, 1:100 (Active Motif Cat# 39155; RRID: AB_2561020); H3K18ac, 1:100 (Active Motif Cat# 39755; RRID: AB_2714186); H3K27ac, 1:100 (Active Motif Cat# 39133; RRID: AB_2561016); H4K20me3, 1:100 (Source BioScience Cat#ABE8663); H4K20me3, 1:50 (Thermo Fisher Scientific Cat# 701777; RRID: AB_2608356)

Secondary antibodies: Goat anti-rabbit (Alexa Fluor 488), 1:300 (Abcam Cat# ab150077; RRID: AB_2630356) and Goat anti-mouse (Alexa Fluor 633), 1:300 (Thermo Fisher Scientific Cat# A-21052; RRID: AB_2535719).

### DNA-FISH

DNA-FISH of PvibSat16-172 satellite specific to the B chromosome was performed following (Larracuente and Ferree 2015; Vea et al. 2025) with minor modifications. Between 10 to 15 testes of males were dissected in 1X DPBS on poly-L-lysine-coated slides, incubated in fixative solution (45% acetic acid (Scientific Laboratory Supplies, Cat#CHE1012) and 4% formaldehyde in 1X DPBS) for 4 min at room temperature, squashed and frozen in liquid nitrogen. Slides were then dehydrated for 10 min in absolute ethanol (Thermo Fisher Scientific, Cat#E/0650DF/08) and air dried. For each slide, 20 ng of the probe (PviSat16-172) was diluted in hybridisation buffer (50% formamide (Thermo Fisher Scientific, Cat#AM9342), 10% Dextran sulphate (Merck Cat#D4911), 1X DPBS), 24 µl of the mixture was deposited on the sample and covered with a coverslip. Slides were denatured at 95°C for 5 min and incubated in a humid chamber at 37°C overnight. After hybridisation, the slides were washed three times in 0.1X SSC (Promega, Cat#V4261) for 5 min at room temperature. After air drying, slides were mounted with DAPI Vectashield mounting medium (Vectashield Cat#H-1200-10).

The probe targeting PvibSat16-172 was previously described (Vea et al. 2025) and is an Oligo probe labelled in 5’ with ATTO488, as follows: 5’-ATTO488CGAGTACGAATGGTAGAAGGT-3’.

### Image acquisition and processing

For both DNA-FISH and immunofluorescence, images were acquired on an upright microscope Leica DM2000 LED with Leica DFC3000 G camera and processed with Leica’s LAS X (RRID:SCR_013673) and Fiji (RRID: SCR_002285) software.

## Supplementary information

Supplementary Information
