# Supplementary material for: Chromatin state dynamics of autosomes and the B chromosome during spermatogenesis in Pseudococcus viburni
Source: Heredity (Edinb). 2025 Oct 13;135(8):597–613. doi: 10.1038/s41437-025-00800-x (PMC13545244; doi:10.1038/s41437-025-00800-x)
Supplement: Supplementary file 1 [file 41437_2025_800_MOESM1_ESM.pdf]

Title

Chromatin state dynamics of autosomes and the B chromosome during spermatogenesis in *Pseudococcus viburni*

Authors

Marion Herbette[1] & Laura Ross[1+]

Supplemental Figure Legends

Figure S1: Schematic representation of paternal genome elimination and spermatogenesis in *P. viburni*. (A) Paternal genome elimination (PGE) in *P. viburni*. Maternal chromosomes are shown in red and paternally inherited chromosomes in blue. In males, paternal chromosomes become heterochromatic and compact (blue circle) during embryogenesis and are eliminated in degrading nuclei after spermatogenesis. In females, both paternal and maternal chromosomes remain euchromatic and undergo recombination during oogenesis. **(B)** Spermatogenesis of *P. viburni*. Structure of the testis organised in units called cysts. Spermatogenesis begins with spermatogonia which undergo four mitotic divisions to form cysts of 16 primary spermatocytes linked by cytoplasmic bridges. These cells then proceed through two meiotic divisions which result in binucleated spermatids, each containing one nucleus with the maternal chromosomes (light gray) and one with the paternal chromosomes (dark gray). Pairs of spermatids fused to form quadrinucleated spermatids. During spermiogenesis, maternal nuclei elongate while paternal nuclei degrade. This results in two sperm bundles, each containing 16 sperm, all carrying the maternal genome.

Figure S2: Immunofluorescence images comparing the effect of different fixation protocols on histone localisation. Testes from second-instar male mealybugs were fixed using three different fixation methods (acid-free fixation, acid fixation and Carnoy's fixation). Acid-free fixation preserves the localisation of histones (red) and H3K27me3 (green) on DNA (blue). No staining is visible outside the nucleus (dashed circle), which is defined using the DNA channel. In contrast, the same anti-H3K27me3 and anti-histone antibodies show weaker signals and altered localisation when samples are fixed with an acetic acid-based fixative (acid fixation) commonly used in squash protocols. In these samples, histones and H3K27me3 are observed both in and outside the nuclei (dashed circle). In addition, H3K27me3 labelling shows variability, either present in the heterochromatin formed by paternal chromosomes (white arrowheads) or absent (yellow arrowheads). This altered labelling pattern is also observed when using Carnoy's fixative (used in previous studies (Bongiorni et al., 2009)), which also alters nuclear and cellular morphology and hardens tissue, making it difficult to obtain good squashes. H3K27me3 and histone labeling are almost completely absent in these samples. Cells shown are in early prophase I. Scale bar: 10 µm.

Fig. S1

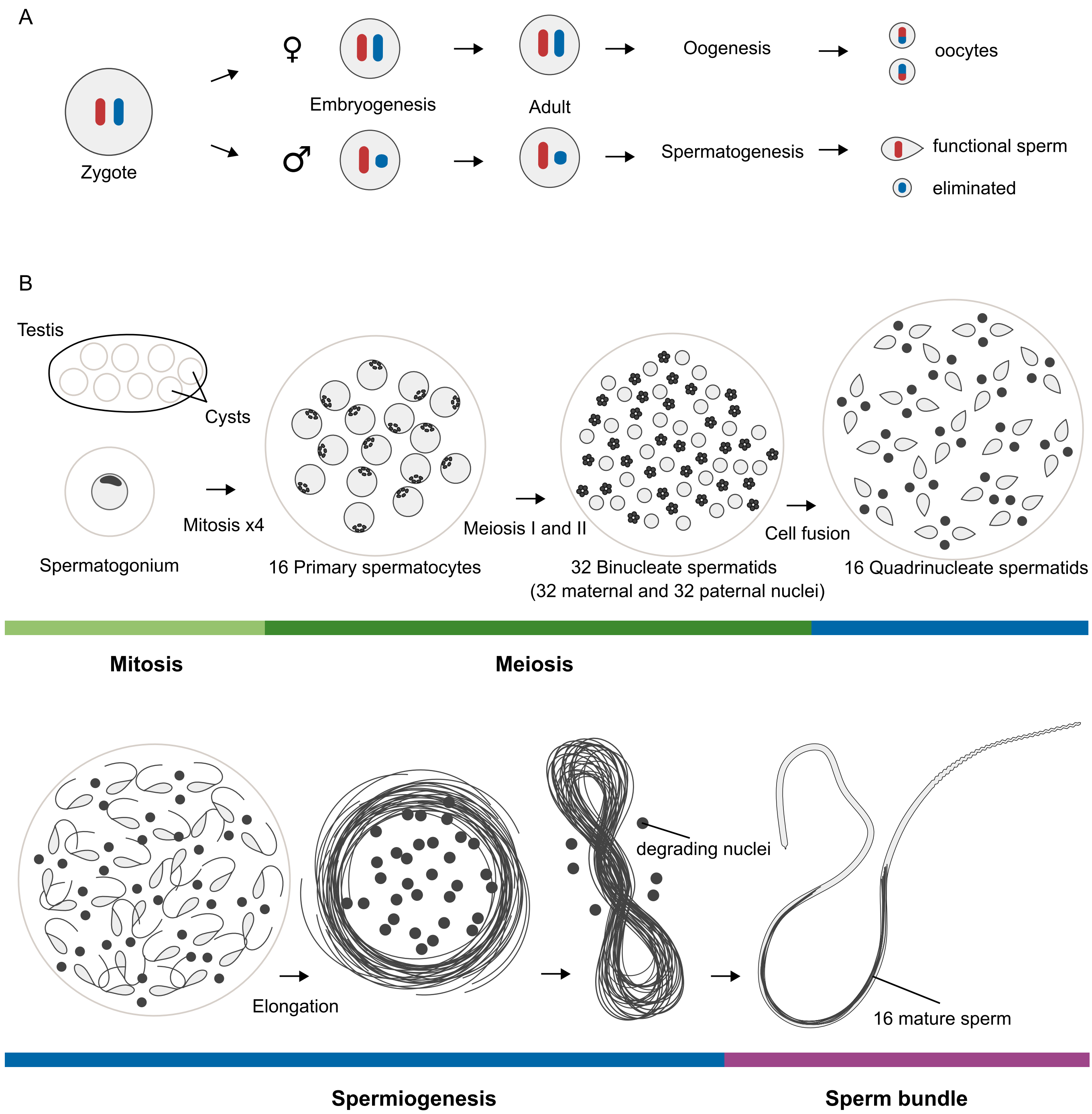
A
Zygote
♀
Embryogenesis
Adult
Oogenesis
oocytes
♂
Spermatogenesis
functional sperm
eliminated
B
Testis
Cysts
Spermatogonium
Mitosis x4
16 Primary spermatocytes
Meiosis I and II
32 Binucleate spermatids
(32 maternal and 32 paternal nuclei)
Cell fusion
16 Quadrinucleate spermatids
Mitosis
Meiosis
Elongation
degrading nuclei
16 mature sperm
Spermiogenesis
Sperm bundle

Fig. S2

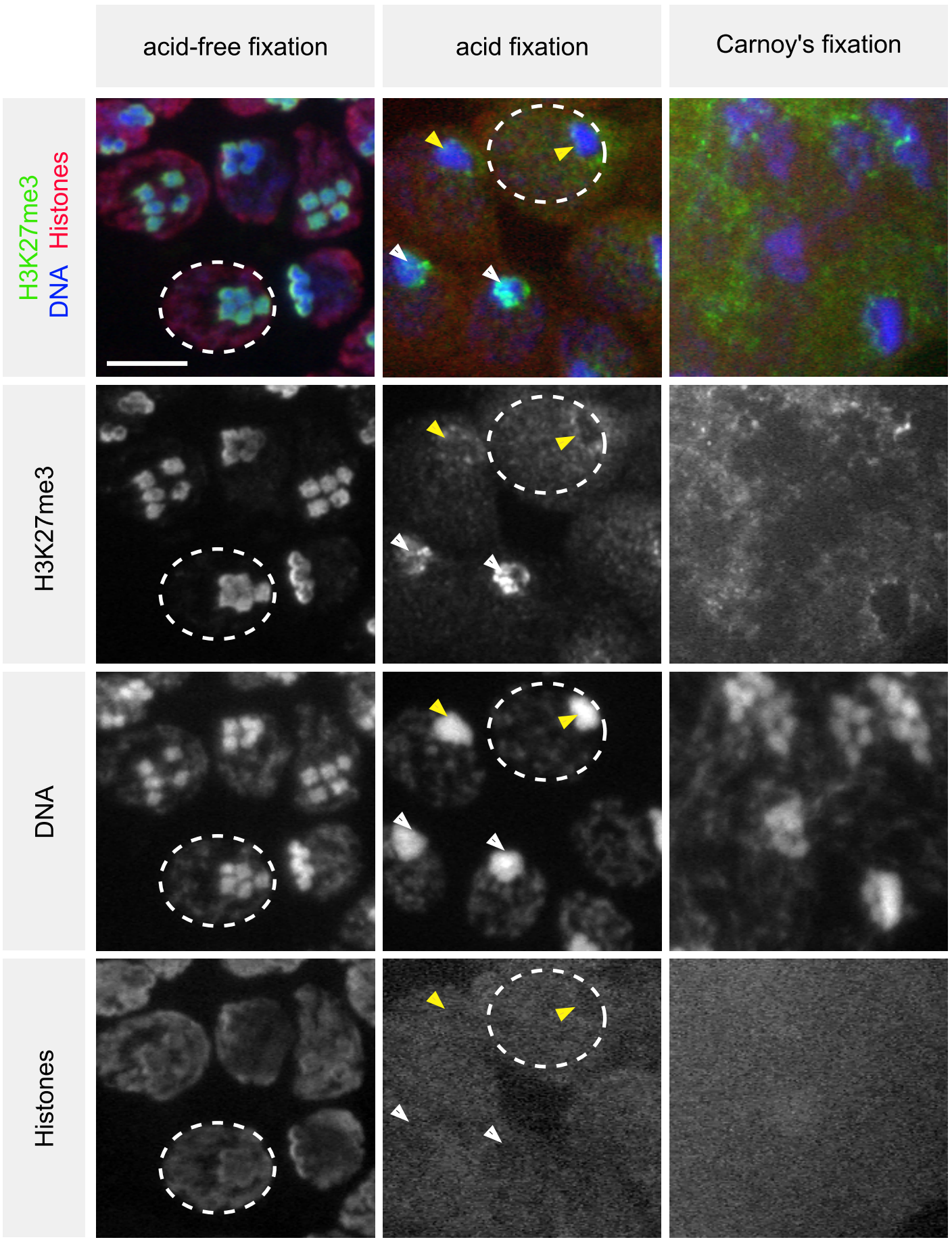

acid-free fixation
acid fixation
Carnoy's fixation
H3K27me3 DNA Histones
H3K27me3
DNA
Histones

Supplemental Methods

Comparison of fixation methods for immunofluorescence on squashed testes

For acid and acid-free fixation, testes were dissected out in PBS-T (1X DPBS, 0.05% Triton) on slides coated with poly-L-lysine. After removing excess liquid, testes were fixed by adding 30µl of 3% paraformaldehyde diluted in 1X PBS (for acid-free fixation) or 3% paraformaldehyde in 45% acetic acid solution (for acid fixation) for 5min at room temperature. A coverslip was placed on top of the sample, then squashed and flash-frozen in liquid nitrogen. The coverslip was quickly removed, and the slides were placed in 100% MeOH for 1min in a Coplin staining jar. For Carnoy's fixation, 2nd-3rd instar males were placed in Carnoy's fixative solution (chloroform, glacial acetic acid, ethanol (4:3:1)) for one hour, then were dissected in 45% acetic acid, squashed and flash freezed in liquid nitrogen. All slides were washed three times for 10min each in PBS-T. The slides were incubated with 30µl of primary antibodies diluted in PBS-T in a humid chamber at 4°C overnight. After three washes of 10min each in PBS-T, the slides were incubated with secondary antibodies and 0.5µg/mL of Hoechst diluted in PBS-T for 2h at room temperature. The slides were washed three times for 10min each in PBS-T and mounted in mounting medium (80% Glycerol, 1X DPBS, 0.2% propyl gallate).

All antibodies were diluted in PBS-T, and are as followed:

Primary antibodies: H3K27me3, 1:100 (Active Motif Cat# 39155; RRID: AB_2561020); Histones, 1:100 (Millipore Cat#MABE71, RRID:AB_10845941).

Secondary antibodies: Goat anti-rabbit (Alexa Fluor 488), 1:300 (Abcam Cat# ab150077; RRID: AB_2630356) and Goat anti-mouse (Alexa Fluor 633), 1:300 (Thermo Fisher Scientific Cat# A-21052; RRID: AB_2535719)
